# Using lignin waste as additives to bitumen for the sustainability of the circular economy: A study of the interaction of mixture components

**DOI:** 10.1371/journal.pone.0350093

**Published:** 2026-06-01

**Authors:** Yuriy Prysiazhnyi, Taras Chipko, Myroslava Donchenko, Roman Serkiz, Viktoria Kochubei, Semen Khomyak, Yuriy Demchuk, Serhiy Pyshyev

**Affiliations:** 1 Department of Chemical Technology of Oil and Gas Processing, Lviv Polytechnic National University, Lviv, Ukraine; 2 Solid State Physics Department, Ivan Franko National University of Lviv, Lviv, Ukraine; 3 Department of Physical, Analytical and General Chemistry, Lviv Polytechnic National University, Lviv, Ukraine; 4 Department of General, Bioinorganic, Physical and Colloidal Chemistry, Danylo Halytsky Lviv National Medical University, Lviv, Ukraine; Oregon State University, UNITED STATES OF AMERICA

## Abstract

The growing role of the circular economy, the shortage of oil, and the expansion of its application areas have led to the search for and use of alternative raw materials that can replace/supplement petroleum products. One direction is the use of lignin, which is produced during cellulose production and can be used as a component/substitute for bitumen binders. Despite significant scientific research in this area, the use of oxidized long-term storage lignin has not been practically studied. Therefore, the purpose of this work was basic research on the interaction between bitumen and lignin, which can later serve as the basis for technologies for the use of this type of waste, obtained in Ukraine and stored for long periods in landfills. The initial lignin and the resulting mixtures with bitumen were characterized using differential thermal and thermogravimetric analysis (DTA/DTG), Fourier transform infrared spectroscopy (FTIR), and scanning electron microscopy (SEM). The results confirm that, despite structural and compositional changes induced by long-term storage, lignin actively interacts with bitumen, forming relatively stable chemical bonds. These results will also be used to develop effective technologies for the utilization of lignin waste accumulated in the Zaporizhzhia region, with direct environmental benefits.

## 1. Introduction

One of the most important tasks of road industry specialists is to improve the performance properties of bitumen binders to increase the service life of asphalt concrete, which is why there is a constant search for and development of new modifiers. At present, a broad range of modifying agents is employed in industrial practice, including styrene–butadiene–styrene (SBS) [1–3], styrene–butadiene elastomer (SBE) [1,4], polyethylene (PE) [1,4,5], polypropylene (PP) [1,6], polystyrene (PS) [7–9], polyvinyl chloride (PVC) [1,5,7,10], atactic polypropylene (APP) [7,8], ethylene butyl acrylate (EBA) [1,7,10], ethylene methyl acrylate (EMA) [1,8,9], ethylene vinyl acetate (EVA) [1,3,10], as well as various organic amines, polyamines, amides, and amido-amines. In addition, the trend of the last 10–20 years is the use of various wastes as additives to bitumen binders: human waste (plastic containers [11–13], used vegetable oil [14–15], waste tires [12–14]); substandard products of various industries (agriculture [11,15,16], processing of hydrocarbon minerals [11–13,17], woodworking [18]); low-quality minerals (sulfur-free and high-sulfur coal [11,13,19]), etc. These applications are designed to enhance bitumen's properties or replace some of it with secondary raw materials, thereby contributing to the growing role of the circular economy and sustainable development.

Despite the type and nature of the substance, its most effective use as a modifier/substitute for part of bitumen is possible only after comprehensive research, in particular, studying the nature of interaction with bitumen binders.

At present, a wide range of analytical techniques is applied to elucidate the interaction mechanisms between potential modifying additives and the base bitumen, as well as to assess their influence on the resulting performance characteristics of the binder. Among the most widely employed methods in such investigations [20–25] are rheological characterization using a dynamic shear rheometer (DSR) and bending beam rheometer (BBR); Fourier-transform infrared spectroscopy (FTIR); modulated differential scanning calorimetry (MDSC); structural group analysis; scanning electron microscopy (SEM); separation into SARA fractions; differential scanning calorimetry (DSC); gel permeation chromatography (GPC); quantum-chemical calculations; nuclear magnetic resonance (NMR) spectroscopy; atomic force microscopy (AFM) for surface topography and microstructural analysis; and complex thermal analysis (DTA/DTG).

It is worth noting that many previous studies have focused on lignin as a partial substitute for bitumen, which affects the binder's performance characteristics [26–31]. In particular, the interaction between lignin and bitumen has been studied; currently, most researchers claim a chemical interaction between the two. However, not all authors have unequivocal confidence in this regard [32,33].

On the other hand, there are many lignin storage facilities for long-term storage. For example, hydrolyzed lignin is stored for more than 30 years in the Zaporizhzhia region (Ukraine) under the influence of atmospheric factors (in the open air). This method of lignin storage obviously has a significant impact on its properties, potentially altering its interactions with petroleum bitumen. Despite the critical scientific research in this area, the use of oxidized lignin for long-term storage has not been practically studied. In previous studies [34,35], the authors investigated the influence of such atypical lignin on the physicochemical characteristics of bitumen binders. In particular, it was found that a bitumen-lignin mixture can be recommended as an analogue of pure bitumen, but lignin must be used in small quantities. At the same time, the nature of the interaction between bitumen and oxidized lignin was not considered. In view of the above, the purpose of this article is to study in detail the mechanisms of interaction between lignin and bitumen binders using complex thermal analysis (DTA/DTG), Fourier transform infrared spectroscopy (FTIR), and scanning electron microscopy (SEM). This integrated approach to studying the interaction between bituminous binders and modifiers has proven effective in our previous studies, particularly in investigating the influence of an organic modifier, namely humic acids extracted from brown coal [34,35]. The feasibility of this study also lies, first of all, in the fact that understanding the mechanism of interaction of lignin with bitumen, ultimately, is of fundamental importance for determining the conditions for the durability of the road surface. For example, oxidized lignin can act only as an inert filler, providing no long-term effect, and can also contribute to the system's phase instability. That is, without understanding the nature of lignin-bitumen interactions, it is impossible to develop technologies for using this type of waste, which is generated in Ukraine and stored for a long time in landfills. Solving the above-mentioned goal of the work will be the main novelty of this research.

## 2. Materials and methods

### 2.1. Materials

The materials used for the experimental studies were technical lignin and bitumen binder (without and with the addition of lignin).

**Fig 1:**
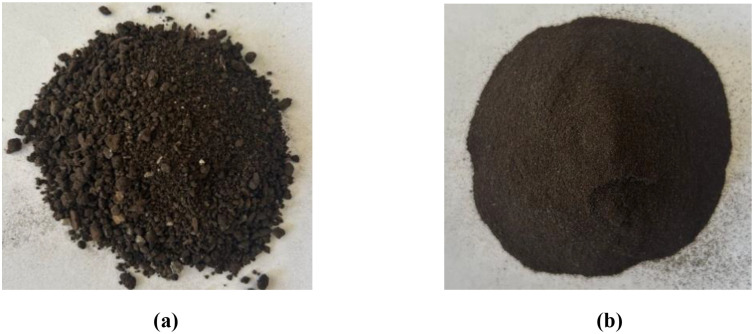
Lignin: (a) collected from the storage; (b) after cleaning from mechanical impurities and drying.

The lignin sample was collected from its long-term storage site in the Zaporizhzhia Region, Ukraine, where no specific permission was required, as the storage and disposal area is publicly accessible. It belongs to the hydrolytic type (obtained using dilute sulfuric acid) and is a non-target product of feed yeast production. Considering the method of securing lignin and its long-term storage, it can be stated that the samples of this raw material are significantly different from the "typical" lignin. The sample contained a significant amount of various undesirable impurities, primarily unprocessed plant material (Fig 1A), as well as products that have accumulated during the storage of lignin in the open air. It also contained inorganic components (slaked lime (Ca(OH)_2_) and gypsum sludge) in the form of small granules or lumps of light color (Fig 1A), which could have gotten into lignin during the production of yeast. In particular, Ca(OH)_2_ is a neutralizer of sulfuric acid during the processing of the hydrolyzate from the hydrolysis of plant material. Gypsum sludge is a non-target product of sulfuric acid neutralization. Using sieves with different mesh sizes, the lignin was classified and purified from unwanted impurities (the yield of pure sample fraction ≤ 0.14 mm according to the material balance was 25.0-30.0% by mass). The resulting fraction ≤ 0.14 mm was then dried to an air-dry state. As a result, a lignin sample was obtained (Fig 1b), the qualitative and quantitative analysis of which is given in Table 1.

**Table 1:** Qualitative analysis of lignin, wt.%.

$W^{a}$	$A^{d}$	$V^{d}$	$\overset{d}{\underset{t}{S}}$	$C^{d}$	$H^{d}$	$N^{d}$	$\overset{d}{\underset{d}{O}}$
2.63^1^	27.45	49.74^2^	0.71	42.36	4.26	0.61	24.61

Notes: $W^{a}$ – the water content relative to the analytical sample; $A^{d}$ – the ash yield relative to the dry sample; $V^{d}$ – the volatiles content relative to the dry sample; $\overset{d}{\underset{t}{S}}$ – the total sulfur content relative to the dry sample; $C^{d}$ – the carbon content relative to the dry sample; $H^{d}$ – the hydrogen content relative to the dry sample; $N^{d}$ – the nitrogen content relative to the dry sample; $\overset{d}{\underset{d}{O}}$ – the oxygen content relative to the dry sample.

When analyzing the data presented in Table 1, it is worth noting the relatively high ash content in the lignin sample under study. It can be assumed that these components will significantly affect the nature of the interaction between lignin and bitumen. However, on the other hand, it is known that various mineral powders (mainly carbonate and silicate) are used in the preparation of asphalt concrete mixtures. At the temperatures at which asphalt concrete mixtures are prepared (over 150 °C), these components do not usually enter into chemical interaction with the binder, but mainly perform a structure-forming function: they fill the pores and compact the structure, ensure the formation of mastic, etc. In view of the above, when analyzing the results obtained regarding the peculiarities of the interaction of lignin with petroleum bitumen, no special attention was paid to the role of ash in this process.

A sample of oxidized bitumen binder grade BND 70/100 was obtained from the oil refinery of PJSC Ukrtatnafta (Kremenchuk, Ukraine) and provided by the manufacturer as part of its cooperation with Lviv Polytechnic National University. The physicochemical properties of the base bitumen, as well as those of the lignin-modified compositions, are presented in Table 2.

**Table 2:** Physical and mechanical characteristics of bitumen.

Index	Unit of measurement			Value
		BND 70/100	Requirements to BND 70/100 according to DSTU 4044:2019 [36]	BND 70/100 + lignin 2 wt.%
Penetration at 25 °C, (P25), measurement error ±2	0.1 mm	78	71-100	70
Softening point (SP), measurement error ±1	°С	52.6	45-51	54.4
Ductility at 25 °C, (D25), measurement error ±4	cm	58	60	34
Elastic recovery at 25 °C, (E25), measurement error ±2	%	17.5	Not applicable	27.5
Solubility in organic solvent	%	99.95	≥ 99.0	–
Adhesion to gravel, measurement error ±0.5	mark	3.5	Not applicable	3.5
Adhesion to glass, measurement error ±5	%	65	≥ 18	58
Resistance to hardening at 163 °C (RTFOT method):				
mass change, (Δm), measurement error ±0.05	wt. %	0.086	Not applicable	0.143
softening point (SP) after RTFOT, measurement error ±1	°С	59.6	Not applicable	60.0
penetration at 25 °C (P25) after RTFOT, measurement error ±2	0.1 mm	39	Not applicable	45
softening point change, (ΔSP)	°С	6.8	Not applicable	5.6
retained penetration, measurement error ±1	%	50.0	Not applicable	64.3

As shown in Table 2, lignin increased the softening temperature, while plasticity (as measured by penetration and ductility) decreased. The adhesiveness of bitumen after adding lignin to it remained practically unchanged, and elasticity improved slightly. Analyzing the change in the properties of bitumen after heating (RTFOT method), we can conclude that lignin to some extent acts as an aging inhibitor: the residual penetration increases and the change in the softening temperature decreases, which is obviously due to the phenolic components of lignin, which can interact with free radicals formed during oxidative aging of bitumen, as described in [37,38]. On the other hand, there is an illogical increase in the mass loss of the bitumen sample modified with lignin (compared to the original bitumen). This increase in mass loss may result from the fact that during the RTFOT analysis process, moisture is completely removed from lignin, which was not completely released during the bitumen modification process. In view of this, the recommendation for the practical use of lignin in the field under study is the need to remove moisture from it before adding it to bitumen; its mass fraction should not exceed 0.1%.

### 2.2. Experimental procedure

#### 2.2.1. Preparation of modified binders.

The modification of bitumen binder with technical lignin was performed under the following experimental conditions:

- process temperature – 120 °С;
- the amount of lignin – 2.0 wt.%;
- process duration – 60 min;
- mixing intensity (mixer Daihan Scientific HT-50 DX) – 1000 min^-1^.

The specific numerical values of the modification parameters were selected based on previously reported studies, particularly those presented in Refs. [35,39,40]. The temperature of mixing lignin with bitumen (120 °C) was chosen to reduce energy costs when implementing the process; this temperature exceeds the softening temperature of bitumen by 67 °C, ensuring homogeneity of the material. It should be noted that a lignin content of 2.0 wt.% in bitumen was identified as optimal in terms of its influence on the key performance characteristics of the binder. In particular, this amount of lignin for the bitumen sample under study is the maximum permissible amount, since a further increase in the amount of this additive may result in the bitumen no longer meeting the requirements of grade BND 70/100: the penetration value may be less than 70 × 0,1 mm. Accordingly, the sample containing 2.0 wt.% lignin was selected for comprehensive thermal analysis and infrared spectroscopic characterization. For scanning electron microscopy (SEM) observations, however, the lignin content was increased to 12.0 wt.% in order to enhance the visual contrast and maximize the ability to assess the effect of lignin on the microstructure of bitumen binder. It is acknowledged that such a substantial increase in lignin concentration relative to the optimal 2.0 wt.% may partially distort its actual structural influence and promote the aggregation of solid lignin particles. Nevertheless, the primary objective of this work was to elucidate the fundamental nature of the interaction between lignin waste and bitumen – specifically to verify or refute the hypothesis of their chemical interaction [41–43] – and to compare the microstructural features of the base bitumen with those of its lignin-modified system. It should be noted that micrographs of a bitumen sample modified with 2.0% lignin by mass were also obtained, but they did not differ from the micrographs of the original bitumen sample.

The modification procedure involved heating the bitumen to the prescribed modification temperature under continuous stirring, followed by the introduction of a predetermined amount of lignin and further aging for the specified duration with constant agitation.

#### 2.2.2. *Methods of analysis.*

Comprehensive thermal analysis of the bitumen and lignin samples was performed using a Paulik–Paulik–Erdey Q-1500 derivatograph coupled with a personal computer for data acquisition. The experiments were carried out in an air atmosphere with heating up to 400 °C at a constant rate of 5 °C min^-1^. The sample mass for each measurement was 200 mg, and aluminum oxide was used as the reference material.

The microstructural features of the bitumen and lignin samples were examined by scanning electron microscopy using a REMMA-102–02 scanning electron microanalyzer (SEMM). The instrument enables nondestructive analysis of both bulk and microscale solid-phase samples, either specially prepared or in their native state. Surface imaging was conducted by scanning with a focused electron beam of several nanometers in diameter at accelerating voltages ranging from 0.2 to 40 kV. The magnification range was 10–300,000 × , with a nominal spatial resolution of approximately 5.0 nm according to the instrument specifications. It should be noted that in order to reduce the ambiguity of the SEM analysis results, the comparison was carried out using micrographs obtained under the same shooting parameters and the same magnification (for the initial bitumen and lignin-modified bitumen, images with the same scales are given: 10 and 20 μm). In addition, the same conditions for sample preparation, the same shooting conditions, and the same criteria for selecting representative areas for analysis and comparison were ensured.

Fourier-transform infrared (FTIR) spectra of the samples were acquired using a PerkinElmer Spectrum Two FT-IR spectrometer equipped with a Universal Attenuated Total Reflectance (UATR) accessory with a diamond crystal, over the spectral range of 4000–400 cm ^-1^. The assignment of IR absorption bands was carried out in accordance with the literature data [44–47]. Baseline correction was applied to all recorded spectra, and the intensities of the absorption bands were determined relative to the baseline. For semi-quantitative analysis and comparative evaluation, the band intensities were normalized to relative values by setting the total sum of all band heights to 100%. Thirteen characteristic absorption bands were selected for reliable interpretation, each associated with specific types of atomic vibrations and chemical bonds. The cumulative contribution of these selected bands accounted for 66–82% of the total normalized spectral intensity.

The main physical and mechanical properties of the original and modified bitumen were determined according to standardized methods. In particular, the following were determined: penetration [48]; softening point [49]; elastic recovery at 25 °C and ductility at 25 °C [50]. Adhesion to the surface of the glass was assessed according to the Ukrainian standard test method DSTU 9169:2021 [51], while adhesion to the gravel surface according to the DSTU 8787:2018 [52] standard. To study the resistance of bitumen to technological aging, the RTFOT method [53] was used.

## 3. Results and discussion

**Fig 2:**
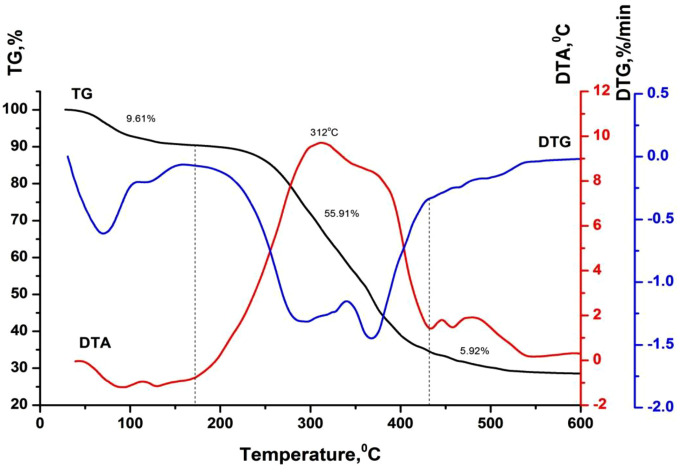
Lignin thermogram.

**Fig 3:**
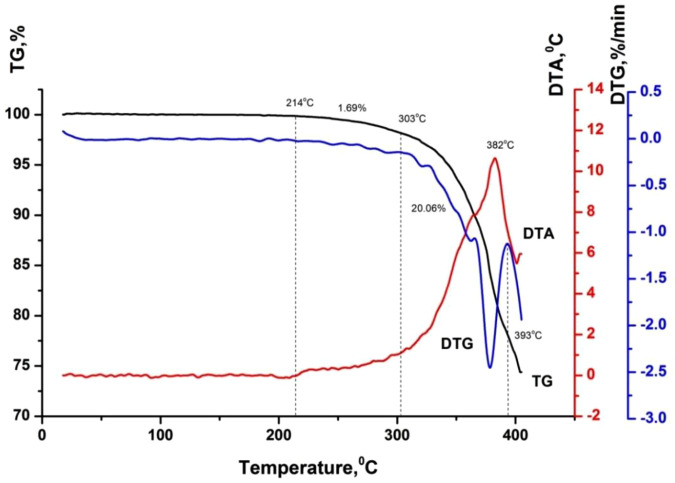
Thermogram of bitumen BND 70/100.

**Fig 4:**
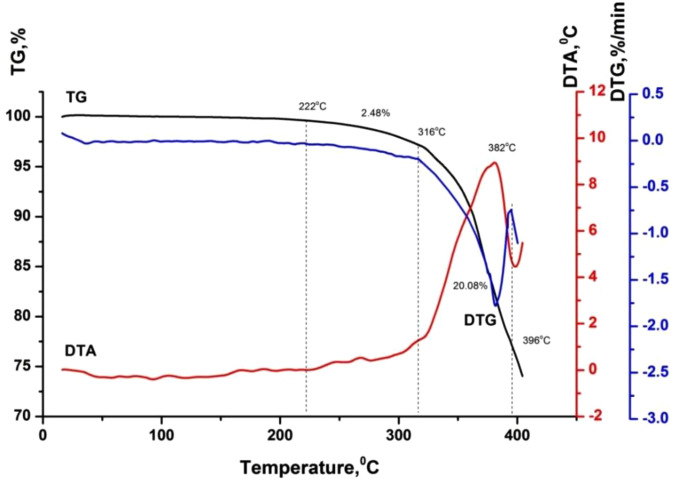
Thermogram of bitumen BND 70/100 + lignin.

**Fig 5:**
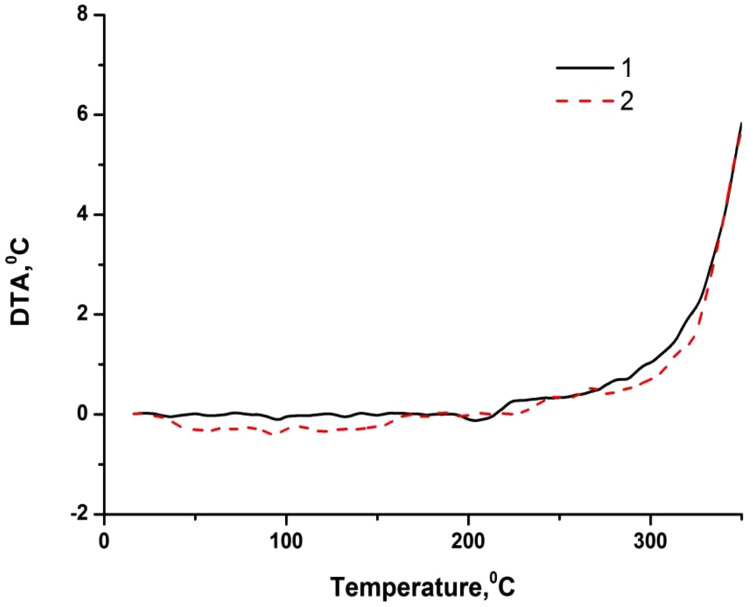
Comparison of DTA curves of samples of original and modified bitumen: 1 – BND 70/100 bitumen, 2 – BND 70/100 bitumen + lignin.

Table 3 and Figs 2-5 show the results of a comprehensive thermal analysis of lignin and lignin modified bitumen samples.

**Table 3:** Results of comprehensive thermal analysis.

Sample	Temperature range, °С	Weight loss, wt.%
Lignin	20–172	9.61
	172–432	55.91
	432–600	5.92
BND 70/100	20–214	0.00
	214–303	1.69
	303–393	20.06
BND 70/100 + lignin	20–222	0.00
	222–316	2.48
	316–396	20.08

The mass loss of the lignin sample amounting to 9.61% in the temperature range of 20–172 °C is attributed to the removal of physically bound moisture and the decomposition of the most thermolabile components (Fig 2). This process is accompanied by the appearance of an endothermic effect on the DTA curve. A pronounced mass loss of 55.91% observed in the temperature interval of 172–432 °C corresponds to the development of thermo-oxidative and destructive transformations, accompanied by the combustion of degradation products. In this temperature region, the DTA curve exhibits an intense exothermic peak with a maximum at 312 °C. At temperatures above 432 °C, the combustion of the lignin pyrolytic residue occurs, which is manifested by a gradual mass loss of 5.92% and a corresponding exothermic effect on the DTA curve Fig 3.

In the temperature range up to 214 ºС, the mass loss of the starting bitumen is not observed, since it is a product of processing heavy oil fractions, which boil off at temperatures above 450–500 ºС. Also, up to 222 ºС, there is no loss of bitumen mass + 2% wt. of lignin, although for pure lignin in this range, the mass loss is about 10% wt. Considering the lignin content in the mixture, the mass loss should be at the level of 0.2% wt. Therefore, it is possible to predict the interaction of the least thermochemically stable lignin components with bitumen during their mixing/heating. A weak endothermic effect is observed on the DTA curve of the lignin-modified bitumen sample within the temperature range of 75–200 °C, which is not accompanied by the release of volatile components (Fig 4). This thermal event is attributed to the softening of the modified bitumen. A gradual mass loss of 2.48% in the temperature interval of 222–316 °C, accompanied by a deviation of the DTA curve toward the exothermic region, corresponds to the onset of thermo-oxidative processes in the sample. Subsequently, a rapid mass loss of 20.08% occurring in the range of 316–396 °C is associated with intense thermo-oxidative degradation, which is manifested by a pronounced exothermic peak with a maximum at 382 °C and a distinct extremum on the DTG curve. At this stage, the maximum rate of mass loss reaches 1.8% per minute.

Based on the results of the comprehensive thermal analysis, it can be concluded that the lignin-modified bitumen exhibits enhanced heat resistance and thermal stability compared to the unmodified binder. The softening of the lignin-modified bitumen in the low-temperature region is accompanied by a weak endothermic effect, which is indicative of the formation of a more compact and structurally ordered system during the modification process (Fig 5).

**Fig 6:**
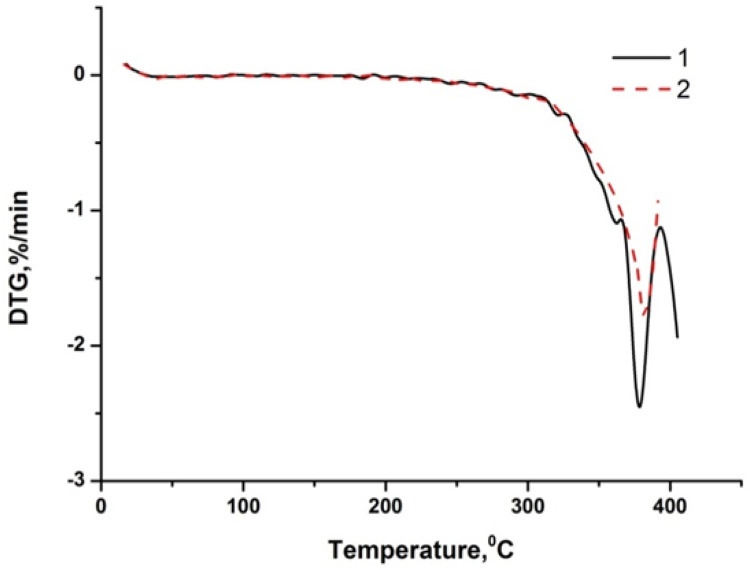
Comparison of DTG curves of samples of original and modified bitumen: 1 – bitumen BND 70/100, 2 – bitumen BND 70/100 + lignin.

Thermo-oxidative degradation of the lignin-modified bitumen initiates at a higher temperature (222 °C) than that of the unmodified bitumen (214 °C). Moreover, these processes in the modified sample proceed at a lower mass-loss rate compared to the original bitumen (Fig 6). The maximum rate of mass loss for the lignin-modified bitumen is 1.8% min ^-1^, whereas for the unmodified binder it reaches 2.5% min ^-1^. The increased thermal stability of the lignin-modified bitumen relative to the base material is further evidenced by the presence of a weak endothermic effect during softening at low temperatures. With further temperature increase, the enhanced thermal stability may be attributed to two main types of processes:

- Bitumen may undergo chemical interaction with lignin, leading to the formation of new compounds with a more compact structure and, consequently, higher thermal stability compared to unmodified bitumen;
- as a result of intensive dispersion within the bitumen matrix, solid lignin particles may form complex structural entities (micelles) with individual hydrocarbons of bitumen or their groups through physical intermolecular interactions, which are also characterized by enhanced thermal stability relative to the base binder.

**Fig 7:**
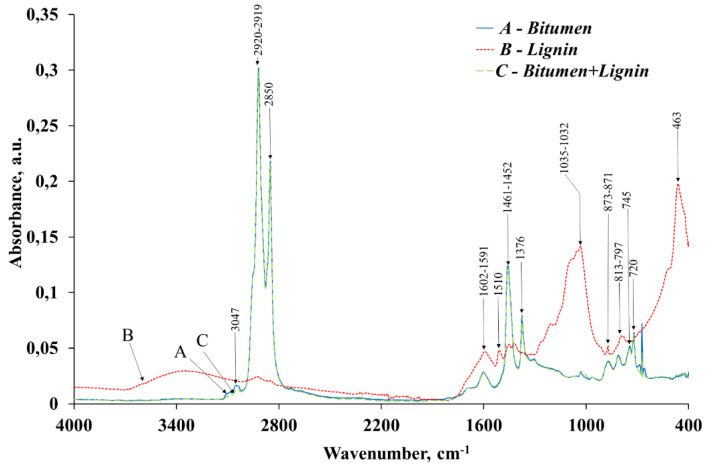
IR spectra: (А) – bitumen BND 70/100; (B) – lignin; (C) – bitumen BND 70/100 + lignin.

To verify or refute these proposed mechanisms, infrared spectroscopic investigations were carried out. The corresponding results are presented in Fig 7 and Table 4.

**Table 4:** Characteristic differences and typical values of peaks of infrared spectra of samples of BND 70/100 bitumen, lignin, and BND 70/100 bitumen modified with lignin.

Wavenumber, cm^-1^	Bitumen		Lignin		Bitumen+Lignin		Structural fragment of the molecule	Atom groups
	Absorbance, a.u.*	Relative %	Absorbance, a.u.*	Relative %	Absorbance, a.u.*	Relative %		
3047	0.0051	0.53	–	–	0.0014	0.15	СН_3_	ν(С–Н) asymmetric
2920−2919	0.2897	29.92	0.0060	1.22	0.2873	30.51	–CH_2_–	ν(С–Н) asymmetric
2850	0.2055	21.22	0.0037	0.76	0.2040	21.66	СН_3_ and СН_2_ or −O − CH_3_	ν(С–Н) symmetric
1602–1591	0.0102	1.05	0.0265	5.36	0.0140	1.48	Ar	ν (С=С) aromatic ring
1510	–	–	0.0212	4.29	–	–	−O − CH_3_ or Ar	δ (CH_3_) methoxyl group or ν (C=С) aromatic ring
1461–1452	0.0950	9.81	0.0214	4.34	0.1001	10.64	-C-H (CH_3_) -C-H(CH_2_) or Ar	δ (CH_3_) asymmetric δ (CH_2_) asymmetric or ν (C=С) aromatic ring
1376	0.0446	4.61	–	–	0.0524	5.57	-С-Н (СН_3_)	δ (СН_3_) symmetric
1035–1032	0.0074	0.77	0.1135	22.98	0.0095	1.01	−O − CH_3_	δ (C–O) methoxyl group
873–871	0.0174	1.80	0.0099	2.00	0.0166	1.76	Ar	δ (C–Н) in aromatic ring
813–797	0.0226	2.33	0.0110	2.22	0.0214	2.27	Ar (–CН = СН–)	δ (C–Н) outside of the area of aromatic ring (mainly in the presence of alkyl substituents)
745	0.0305	3.15	–	–	0.0286	3.04	Ar (–CН = СН–)	δ (C–Н) outside of the area of aromatic ring (mainly in the presence of alkyl substituents)
720	0.0427	4.41	–	–	0.0410	4.36	Ar (–CН = СН–)	δ (C–Н) outside of the area of aromatic ring (mainly in the presence of alkyl substituents)
463	–	–	0.1144	23.17	–	–	Me(СН_3_)x	ρ (Me-C)
Total	0.7707	79.60	0.3276	66.33	0.7763	82.45	–	–
The rest of the IR bands	0.1976	20.40	0.1663	33.67	0.1653	17.55	–	–

*a.u. – absorption units; δ – deformation oscillations; ν – valence oscillations; ρ – rocking vibration.

**Fig 8:**
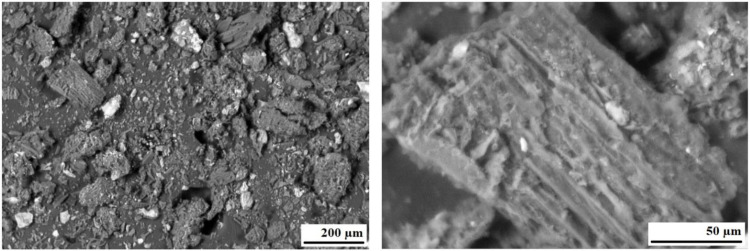
Micrographs of lignin.

**Fig 9:**
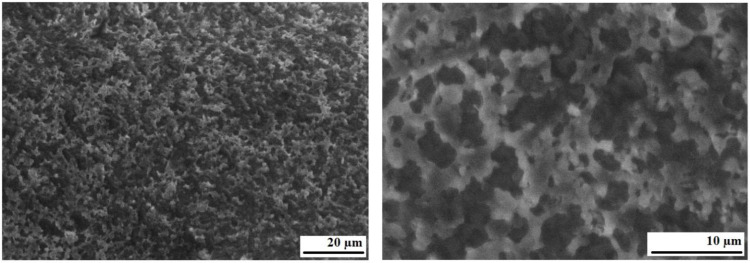
Microphotographs of bitumen BND 70/100.

**Fig 10:**
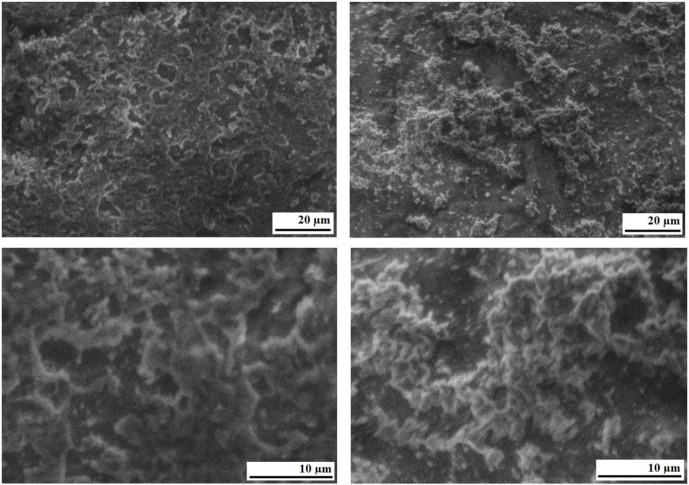
Microphotographs of bitumen BND 70/100 + lignin.

The IR spectroscopic analysis indicates that the bitumen matrix comprises aromatic, aliphatic, and cycloalkane structural fragments. The aromatic structures of bitumen (unlike lignin) contain side chains (peaks 813–745 cm^-1^). The most distinct absorption bands of lignin are the peaks corresponding to methoxyl groups (absent in bitumen), which are consistent with similar studies [46,47]. Additionally, the lignin under study may contain organometallic compounds (peak at 463 cm^-1^). Given the long-term storage of lignin in the open air, the presence of these organometallic substances is explained by the possible interaction of lignin and mineral clays or waste, as well as the above-described method of obtaining lignin. It should also be noted that the “untypicality” of the used lignin waste is confirmed by the presence of some very noticeable peaks (for example, 463, 1035–1032 cm^-1^), which are absent or noticeably smaller in similar studies [45,47,54,55]. After the incorporation of lignin into the bitumen matrix, the disappearance of several absorption bands associated with reactive methoxyl groups and organometallic structures (at 1510 and 463 cm ^-1^) is observed. This spectral change suggests the occurrence of partial chemical interaction between lignin and bitumen under the applied mixing conditions. If so, the mixture of lignin (solid) and bitumen (liquid) should be a homogeneous system, at least partially. To confirm this, microphotographs of bitumen and lignin samples were obtained by scanning electron microscopy (Figs 8–10).

Fig 8 shows micrographs of technical lignin, which indicate that the sample used consists of particles with a maximum size of less than 200 μm. At the same time, its main mass is a finely dispersed material with particle sizes of 10–50 μm. Based on their surface structure, the particles studied can be divided into two types: variable configuration with sizes of 10–20 μm and elongated (20–30 μm thick and 50…150–200 μm long), characterized by a fibrous surface. The former belong to the inorganic phase, while the latter are apparently destroyed organic fibers remaining after the extraction of lignin from wood. It should be noted that the ash content of the investigated sample, amounting to 27.45 wt.% (Table 1), confirms the significant presence of inorganic constituents. Also, the main part of the sample has a smooth surface, the amount of which can be estimated at 40–50%. Since lignin is an amorphous substance, it can be argued that this is, in fact, the lignin phase.

Fig 9 shows micrographs of the surface of BND 70/100 bitumen. It should be noted that preparing the sample surface by sputtering and scanning with an electron beam in a microscope chamber involves the use of a deep vacuum and heating. Such conditions obviously lead to the evaporation of the oil components of bitumen. Therefore, it can be argued that the micrographs show a relatively porous phase with pore sizes of 2–5 μm, which obviously consists of a dispersed bitumen medium (asphaltenes and resins). Visually, the porosity can be estimated at 30–40%.

Fig 10 shows that in the bitumen sample modified with 12% lignin by mass, no large lignin particles are visually observed as separate structural units or as large micelles formed as a result of physical intermolecular interactions. In some areas, only small inclusions of lignin particles with a fibrous structure 3–5 μm in length are observed. The surface of bitumen with lignin additives is characterized by a denser structure, with fewer pores, the average size of which is 5–10 microns. The obtained results indicate that the investigated mixture represents a relatively homogeneous system, which can be attributed to the formation of sufficiently strong chemical bonds between the reactive functional groups of lignin (e.g., methoxyl groups) and the functional groups present in bitumen. Thus, the microstructural analysis corroborates the findings of comprehensive thermal analysis and IR spectroscopy, confirming the enhanced heat resistance and thermal stability of lignin-modified bitumen, as well as supporting the plausibility of chemical interaction between lignin and bitumen.

## 4. Conclusions

The results of comprehensive thermal analysis demonstrate that the lignin-modified bitumen exhibits superior heat resistance and thermal stability compared to the unmodified binder. The onset of thermo-oxidative degradation in the modified sample is shifted to a higher temperature (222 °C) than in the original bitumen (214 °C). Moreover, the thermo-oxidative processes in the lignin-modified bitumen proceed at a lower mass-loss rate than in the unmodified sample. The maximum rate of mass loss for the modified bitumen is 1.8% min ^-1^, whereas for the original bitumen it reaches 2.5% min ^-1^.

Infrared spectroscopic analysis revealed that, following the incorporation of lignin into the bitumen matrix, several absorption bands characteristic of reactive methoxyl groups and organometallic structures (at 1510 and 463 cm ^-1^) disappear. In addition, microstructural examination demonstrated that the lignin-modified bitumen contains only minor inclusions of lignin particles with sizes in the range of 3–5 μm. The surface of bitumen with lignin additions exhibits a somewhat denser structure and smaller pores than the original bitumen.

The used analytical methods (complex thermal analysis, Fourier transform infrared spectroscopy, and scanning electron microscopy) allowed us to conclude that modifying petroleum bitumen binder with lignin waste (under selected conditions) is a chemical process. Despite several differences between the studied lignin waste and “typical” lignin, they form bonds with the original bitumen during their heating/mixing.

The results obtained allow us to predict the feasibility of using and the potential for developing an effective technology for lignin waste, which is stored for a long time in the Zaporizhzhia region. This, in turn, will reduce environmental pollution and promote a circular economy by partially replacing bitumen with waste at no cost.

At the same time, the authors recognize the limitations of current research in developing an effective technology for oxidized lignin. Given the possible chemical nature of the interaction between lignin waste and bitumen, observed even at 120 °C, it is necessary to assess further the feasibility and effectiveness of using different lignin additives at elevated temperatures, which will accelerate bond formation. Also, the goal of further research in this direction will be comprehensive studies of the operational characteristics of bitumen-lignin systems: rheological tests (using a dynamic shear rheometer (DSR)), low-temperature tests, modeling of operational aging, and assessment of storage stability.

### Symbols and designations

$W^{a}$ the water content relative to the analytical sample, wt.%

$A^{d}$ the ash yield relative to the dry sample, wt.%

$V^{d}$ the volatiles content relative to the dry sample, wt.%

$\overset{d}{\underset{t}{S}}$ the total sulfur content relative to the dry sample, wt.%

$C^{d}$ the carbon content relative to the dry sample, wt.%

$H^{d}$ the hydrogen content relative to the dry sample, wt.%

$N^{d}$ the nitrogen content relative to the dry sample, wt.%

$\overset{d}{\underset{d}{O}}$ the oxygen content relative to the dry sample, wt.%

## Abbreviations

DTA: Differential thermal analysis
DTG: Derivative thermogravimetry
FTIR: Fourier-transform infrared
RTFOT: rolling thin film oven test
